# Supplementary material for: Density Functional Theory and Density Functional Tight Binding Studies of Thiamine Hydrochloride Hydrates
Source: Molecules. 2023 Nov 9;28(22):7497. doi: 10.3390/molecules28227497 (PMC10673443; doi:10.3390/molecules28227497)
Supplement: Supplementary file 1 [file molecules-28-07497-s001.zip › molecules-2689151-supplementary.pdf]

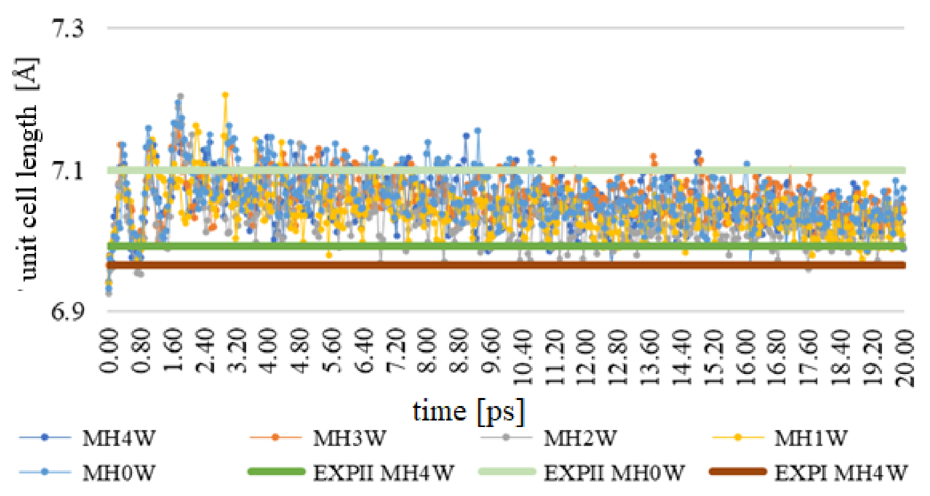

A1 „a” unit cell length of NSH

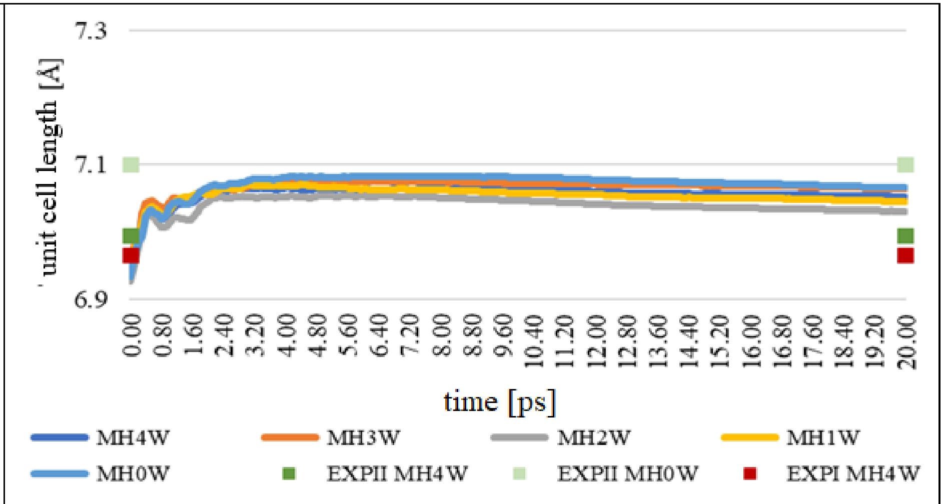

A2 „a” unit cell length of NSH, running average

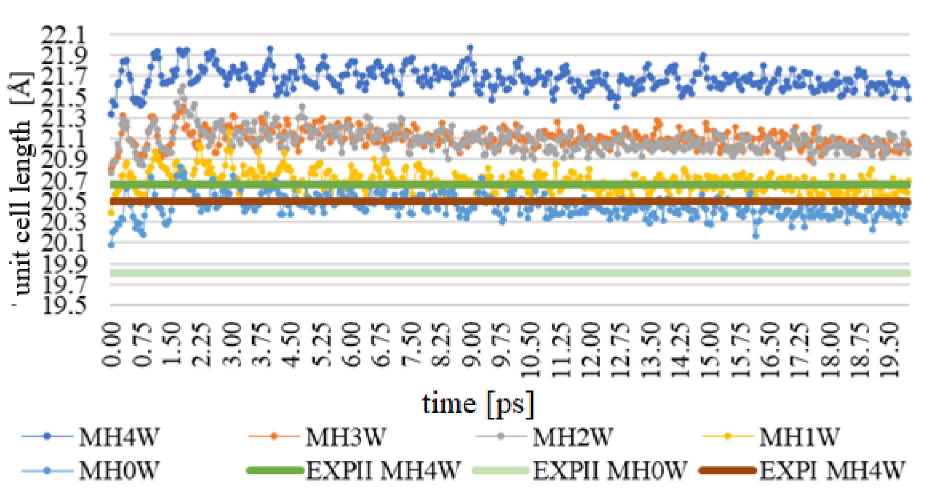

B1 „b” unit cell length of NSH

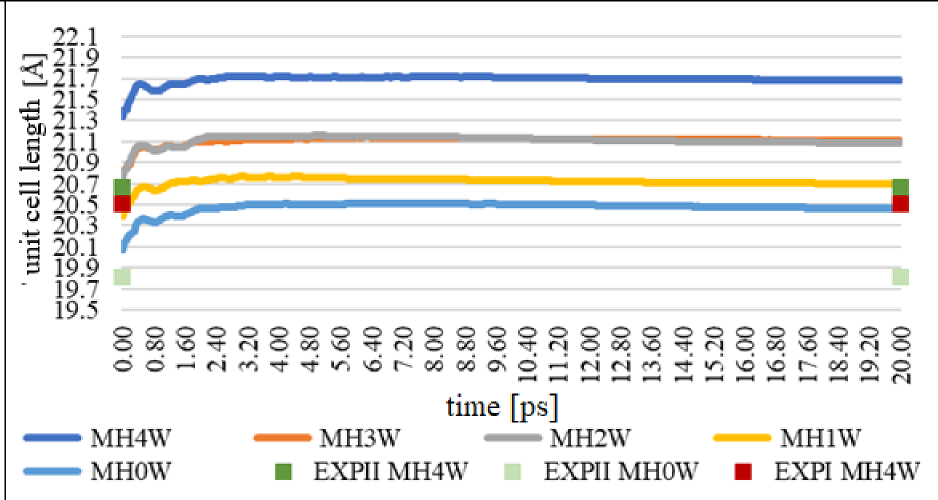

B2 „b” unit cell length of NSH, running average

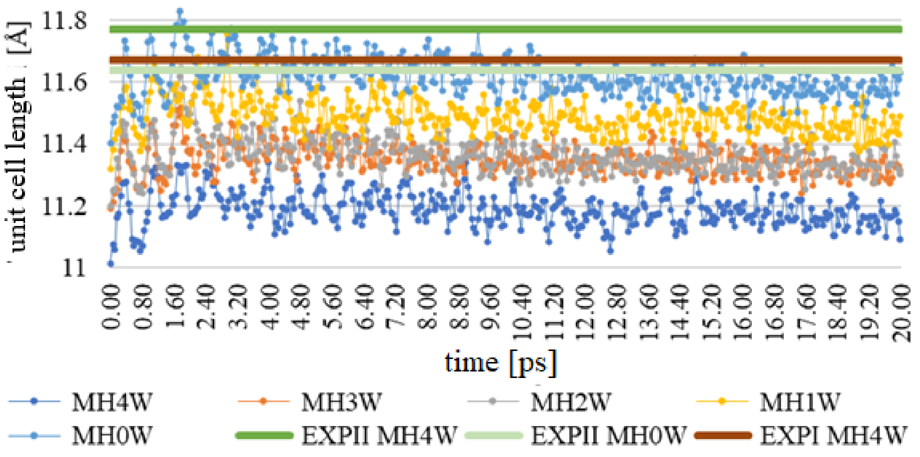

C1 „c" unit cell length of NSH

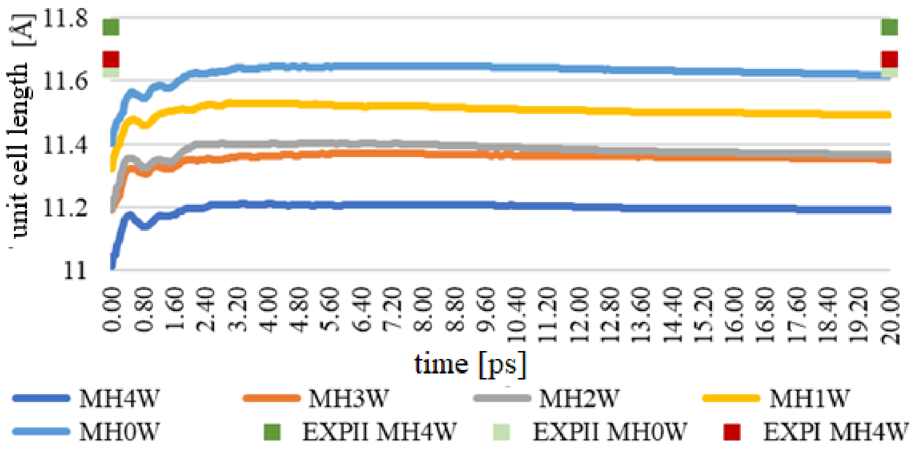

C2 „c" unit cell length of NSH, running average

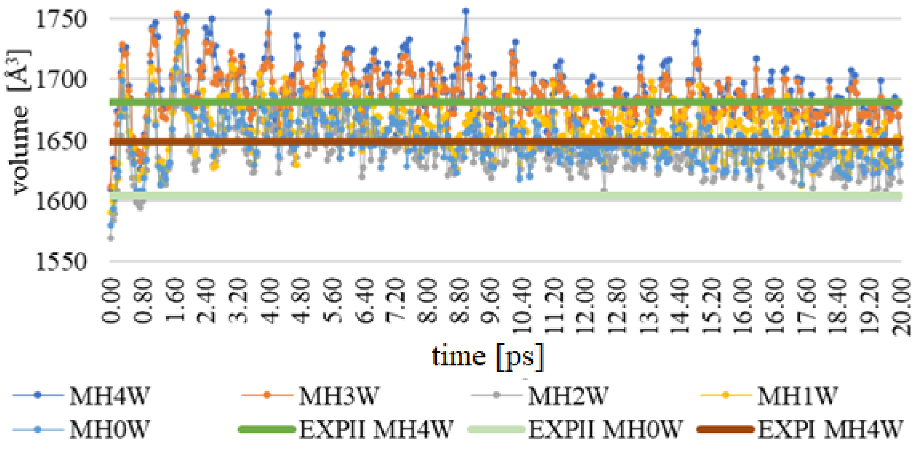

D1 unit cell volume of NSH

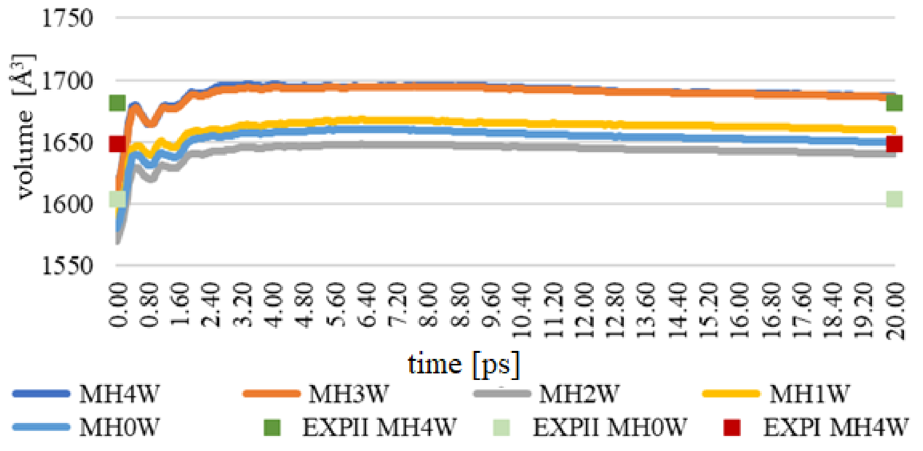

D2 unit cell volume of NSH, running average

Figure S1. Changes in the unit cell dimensions of NSH observed during the molecular dynamics simulations.

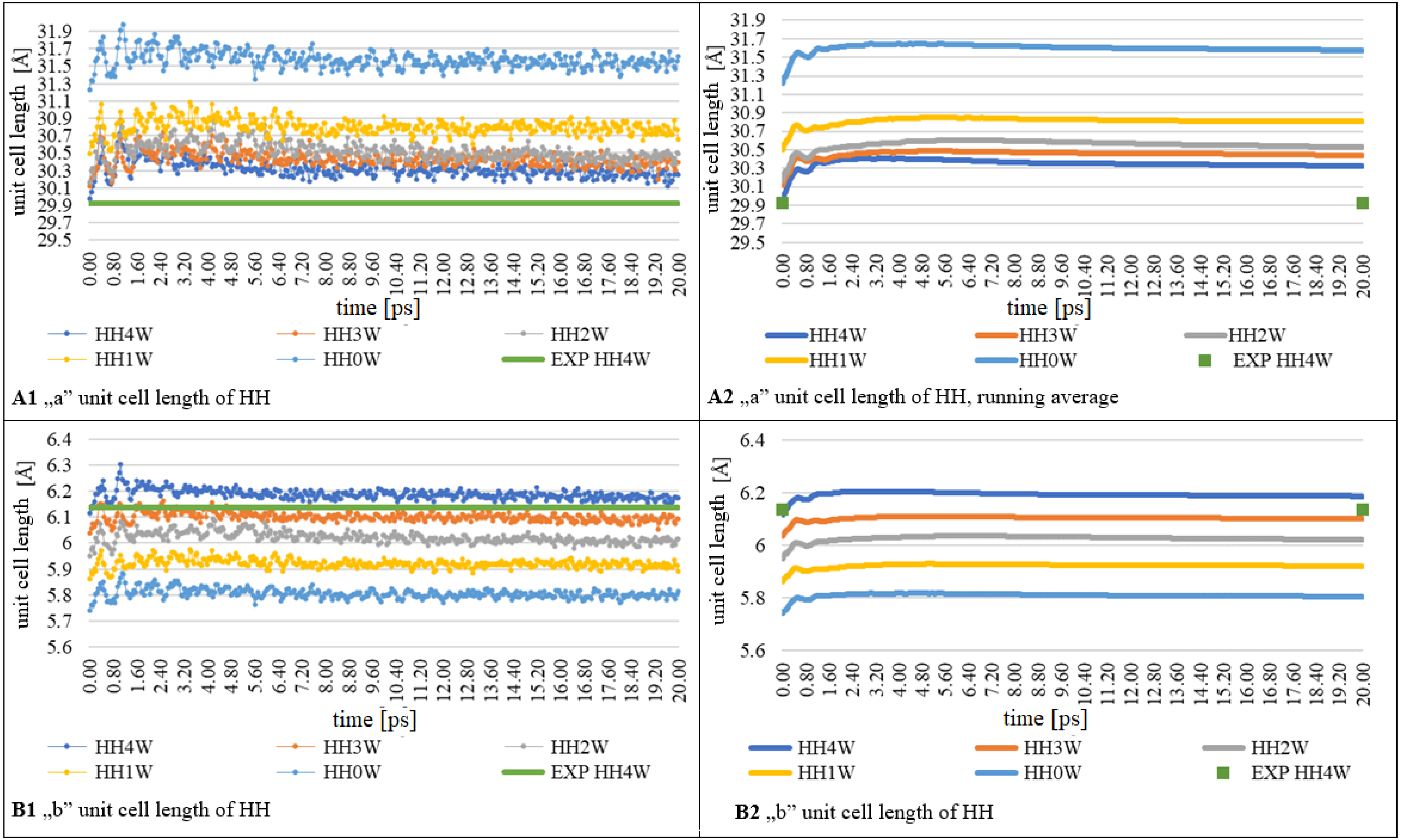

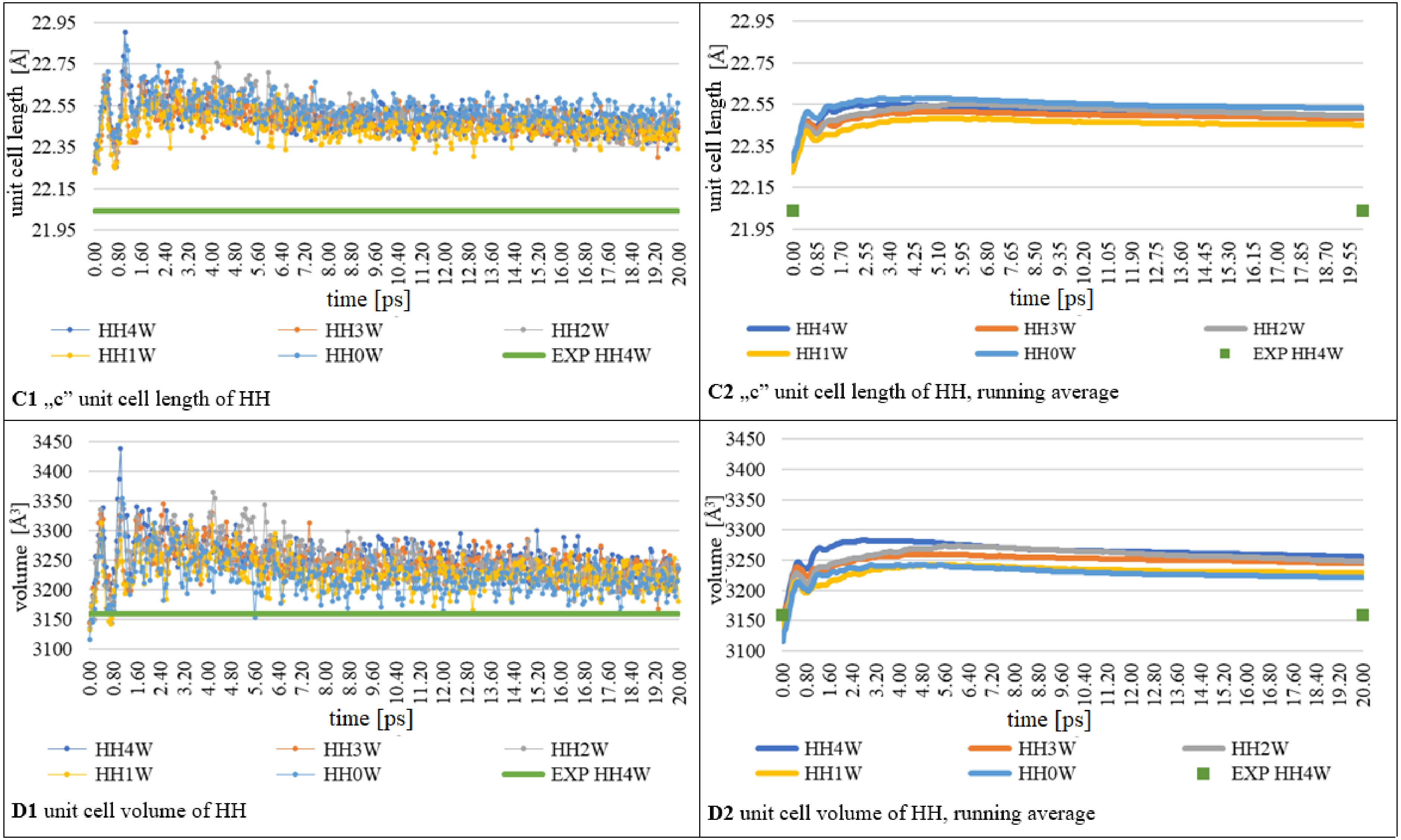

Figure S2. Changes in the unit cell dimensions of HH observed during the molecular dynamics simulations.
